# Research on the Design and Performance of Plant Volatile Organic Compounds Water Removal Device Based on Optimized Filler Ratio

**DOI:** 10.3390/mps7040059

**Published:** 2024-07-31

**Authors:** Yali Yuan, Huasen Wang, Zhihong Sun, Chao Yu

**Affiliations:** 1 Collaborative Innovation Center for Efficient and Green Production of Agriculture in Mountainous Areas, College of Horticulture Science, Zhejiang A&F University, Hangzhou 311300, China; 2 Engineering Laboratory of Genetic Improvement of Horticultural Crops of Shandong Province, College of Horticulture, Qingdao Agricultural University, Qingdao 266109, China; whsych66@163.com; 3 College of Agriculture and Life Science, Liaocheng University, Liaocheng 252059, China

**Keywords:** biogenic volatile organic compounds (BVOCs), water removal device, filler optimization

## Abstract

This study focuses on the development and optimization of a water removal device for biogenic volatile organic compounds (BVOCs) from plant emissions. BVOCs play a crucial role in various ecological processes and have potential therapeutic effects on human health. However, it is challenging to accurately detect and analyze BVOCs due to their very low concentrations and interference by water vapor. This study systematically evaluates different filler materials and ratios to alleviate water vapor interference while maintaining BVOCs’ integrity. The experimental results demonstrate that the combination of MgSO_4_ + Na_2_SO_4_ mixed filling and CuSO_4_ layered filling in a 3:3:1 ratio can effectively improve the collection efficiency and detection accuracy of BVOCs. Meanwhile, the effectiveness of the device in improving the detection of volatile compounds in plant samples is also confirmed by the VOC verification experiments on *Michelia maudiae* and *Cinnamomum camphora* tree species after mechanical damage. The experimental results show that the device is effective in improving the detection of volatile compounds in plant samples. The findings provide a powerful technical means for exploring the role of BVOCs in environmental monitoring and scientific research.

## 1. Introduction

Volatile organic compounds (VOCs) are a category of small molecules primarily composed of carbon, hydrogen, and oxygen, known for their high volatility. Although VOCs are widespread in the atmosphere, their concentrations are typically low, often measured in parts per billion (PPb) or even parts per trillion (ppt) [1]. These compounds originate from various sources, including emissions from cleaning products [2,3], industrial processes [4], and vehicle exhaust [5], as well as natural sources such as plant emissions [6,7], animal metabolism [8], and microbial activity [6]. It is worth noting that plants are the major contributors, accounting for around 90% of global VOC emissions, with live plants being the primary source [9]. Plants emit a variety of compounds, with forests alone emitting over a hundred different types of BVOCs (biogenic volatile organic compounds), with isoprene, monoterpenes, aromatic compounds, and green leaf volatiles being the most abundant [2,10,11]. These BVOCs serve multiple functions within ecosystems, including plant reproduction [12], defense mechanisms against pests and diseases [8], as well as interactions with other organisms [13,14,15]. Moreover, recent research has highlighted the potential therapeutic effects of plant volatiles on human health, with implications for emotional regulation, stress relief, and even mood disorders [16].

Given the significance of BVOCs, precise detection methods are crucial. However, their low concentrations pose challenges for accurate qualitative and quantitative analysis, and they are subject to interference from water vapor during sampling and analysis processes. Water vapor can hinder the adsorption of BVOCs during gas collection, leading to losses in chromatographic columns, thus affecting the reliability of the analytical results. Additionally, water vapor interference in mass spectrometry analysis can affect the accuracy of qualitative analysis [17].

To solve the problem of water vapor interference in BVOCs monitoring and analysis, this study focused on the development of a simple and efficient BVOCs water removal device. The device was designed to mitigate water vapor interference while maintaining the integrity of BVOCs samples during collection and analysis. In order to achieve this goal, the adsorption properties, stability, and effectiveness of various fillers were comprehensively considered in the study. The effects of different filling ratios on water vapor removal without affecting the adsorption of BVOCs were studied. The effectiveness and reliability of the designed device were further proven by actual verification in practical applications.

## 2. Materials and Methods

### 2.1. Materials

#### 2.1.1. Screening of Dehydrating Fillers

The effects of different dehydrating fillers on the performance of gas volatile removal devices were evaluated, and a large number of research results and practical experiences were integrated to select dehydrating fillers. Studies have shown that anhydrous sodium sulfate (Na_2_SO_4_) is hygroscopic and forms crystalline hydrates when exposed to air [18,19]. It can be easily dehydrated by heating and is easy to reuse, so it is widely used in dehumidifiers and gas treatment equipment. In these research studies, anhydrous magnesium sulfate (MgSO_4_) was identified as a common hygroscopic compound and desiccant that can effectively absorb moisture without chemically reacting with most organic matter [20,21,22]. The crystalline hydrate formed after absorbing water is also easy to dehydrate and reuse, making it a common filler in gas volatile removal devices. Anhydrous copper sulfate (CuSO_4_) is more representative, and is hygroscopic, forming blue hydrates after absorbing water [23]. The color change can be used as an indicator for real-time monitoring, improving the efficiency and monitoring capabilities of the equipment. Therefore, this study selected Na_2_SO_4_, MgSO_4_, and CuSO_4_ dewatering fillers for testing in different combinations and proportions to determine their dewatering effects.

#### 2.1.2. Plant Materials

*Michelia maudiae* (Magnoliaceae) and *Cinnamomum camphora* (Lauraceae) tree species were used as the plant materials in the experiment. These trees were sourced from the East Lake Campus of Zhejiang A&F University (30°15′ N, 119°43′ E), situated in the forest city of the western suburbs of Hangzhou. This area experiences a warm climate with ample rainfall throughout the year. The region boasts a diverse array of plant species, with an average temperature of approximately 28.5 °C and relative humidity levels reaching up to 70%. These selected tree species are characterized by their large scale and high ornamental value, making them ideal candidates for experimental verification.

### 2.2. Experimental Design

The quartz glass tube used in this experiment had an outer diameter of 6.4 mm, an inner diameter of 4.4 mm, a wall thickness of 1 mm, and a length of 85 mm. Quartz glass, known for its excellent corrosion resistance and high temperature stability, was suitable for treating volatile gases in the dewatering device.

#### 2.2.1. Preliminary Comparison of Dewatering Fillers

**Figure 1:**
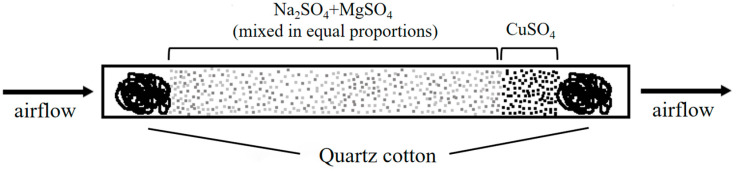
Diagram of the sample tube filling for the water removal device.

To evaluate the removal effect of different dewatering fillers (MgSO_4_, Na_2_SO_4_, MgSO_4_ + Na_2_SO_4_) on the main volatile organic compounds (isoprene, trichloromethane, benzene, toluene, chlorobenzene, p-xylene, pinene) in the standard gas, different treatment groups were defined. The name of the water removal device, the compositions and proportions of experimental fillers in the device, and the source of samples tested by the device are shown in Table 1, and a sample tube filling diagram of the water removal device is shown in Figure 1.

#### 2.2.2. Screening of Water Removal Fillers

The combined filler comprising MgSO_4_ and Na_2_SO_4_ demonstrated the most effective water removal. Furthermore, the water removal efficiency of the combined filler of MgSO_4_ + Na_2_SO_4_ using different filling methods was evaluated. The water removal device with layered filling and mixed filling methods (filled with MgSO_4_ + Na_2_SO_4_) was tested in a forest environment to detect the BVOCs components in the forest.

#### 2.2.3. Optimizing the Dewatering Filler Combination

Combining the best results of the water removal filler in the mixed filling method, the dewatering filler combination was further optimized by adjusting the mixing ratio of MgSO_4_ + Na_2_SO_4_, incorporating CuSO_4_ as an indicator. Different ratios of MgSO_4_ + Na_2_SO_4_ (1:1 and 2:1) were used for mixed filling, and CuSO_4_ was added as an indicator to form DMm(MgSO_4_ + Na_2_SO_4_), DMm1, DMm2, and DMm3 water removal devices. The impacts of these the water removal devices on the types and contents of BVOCs were investigated in the forest settings to determine the optimal ratio of water removal fillers.

#### 2.2.4. Result Verification

Based on the experimental results, the optimal filler combination (mixed filling of MgSO_4_ + Na_2_SO_4_ and layered filling of CuSO_4_ with a ratio of 3:3:1) was selected for verification. The optimal filler combination was used to collect BVOCs from mechanically damaged leaves of *Michelia maudiae* and *Cinnamomum camphora* tree species. BVOCs were collected in a forest environment using a micro individual sampler produced by the Beijing Institute of Labor Protection Science. The flow rate was set to 120 mL/min, and the sampling duration was 30 min. After sampling, thermal desorption/gas chromatography/mass spectrometry (TD/GC/MS) was used for qualitative measurement to analyze the composition of the sampled gas and confirm that the water removal filler removed gas volatiles to further verify the water removal effect.

### 2.3. Measurement and Qualitative and Quantitative Analysis Methods of BVOCs

Thermal desorption/gas chromatography/mass spectrometry (TD/GC/MS) was used to measure and analyze plant volatile organic compounds (VOCs). In the experiment, the operating conditions of the thermal desorption device (TD-100, Markes international, Bridgend, UK) were set to an initial temperature of −5 °C, pre-blow time of 3 min, primary desorption of 5 min, and secondary desorption of 3 min. The cold trap temperature was raised from 40 °C to 280 °C and held for 3 min, and the split ratio was 20:1. The gas chromatograph (GC, 7890B, Agilent, Agilent Technologies, Santa Clara, CA, USA) used a DB-624 Agilent, 60 m length, 0.25 mm inner diameter, and 0.25 μm film thickness apillary column, with a programmed temperature rise of 40 °C (hold for 5 min) → 4 °C/min → 150 °C (hold for 3 min) → 10 °C/min → 220 °C. The mass spectrometer (MS, 5977B, Agilent, USA) used the EI source with an ion energy of 70 eV, ion source temperature of 230 °C, quadrupole temperature of 150 °C, mass range of 35–260 *m*/*z*, and interface temperature of 280 °C.

For structural analysis, the mass spectra were collected using MassHunter workstation software (version B.07.00, Agilent Technologies, USA), and the molecular formula and structural formula of the compounds were determined by searching the NIST Chemistry WebBook (National Institute of Standards and Technology, Gaithersburg, MA, USA, NIST 14) library. After the volatile components were identified, they were calibrated using standard gas produced by Ionicon. The standard gas contained 15 compounds with a volume mixing ratio of approximately 1.00 ± 0.05 ppm, and the mixing ratio of the standard gas to pure nitrogen was 1:49. After mixing and passing through the open pipeline for a period of time, the gas collection device was connected; the flow rate of the sampling pump was set to 100 mL/min; samples were collected for 30 min, 40 min, 50 min, and 70 min. At the same time, each gradient was collected by the same method with pure nitrogen as a control. Each gradient sample was collected 3 times. Finally, a standard curve was drawn based on the mass spectrometry peak area and compared with the collected standard gas volume, and the peak area of the corresponding substance in the sample was extracted for qualitative and quantitative analysis. Detailed and specific methods were as described by Yuan et al. [24].

### 2.4. Data Analysis

All experiments were arranged in an independent, complete design with three replicates. The values presented are the means ± SE of the three replicates. The preliminary data collection for this experiment was conducted using Microsoft Excel 2017. Subsequently, statistical analyses were conducted using analysis of variance (ANOVA) with SPSS 22.0 software. Differences between treatments were determined by least significant differences with *p* < 0.05. Differences in average BVOCs emission rates, with and without a water removal device, were also analyzed using paired-sample *t*-tests. The results were visualized using Origin 2021 plotting software.

## 3. Results

### 3.1. Preliminary Comparison of the Water Removal Effects of Different Filler Combinations

**Figure 2:**
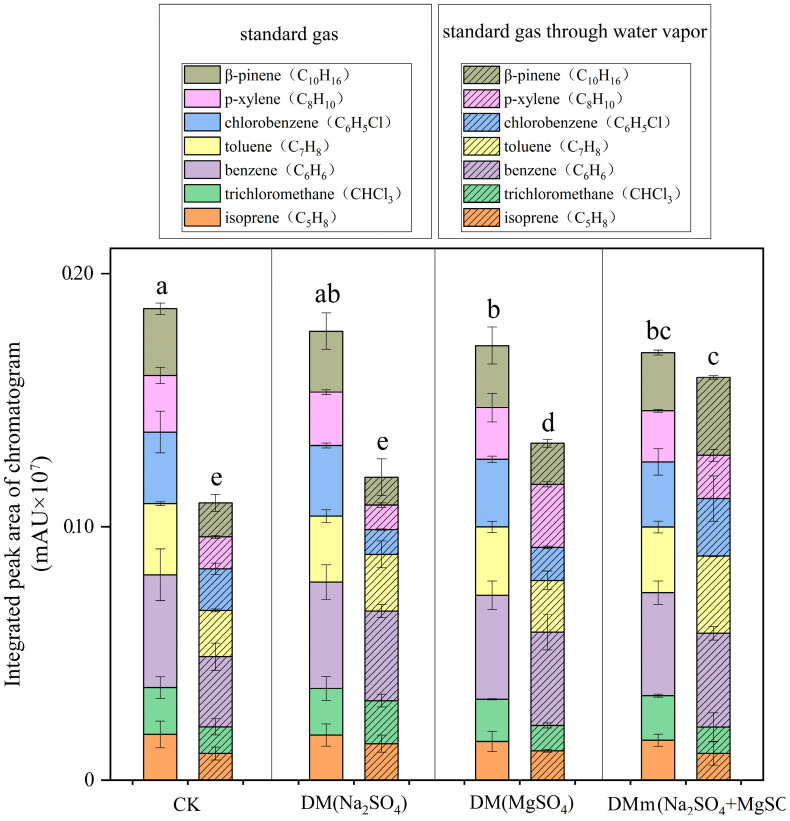
Effects of types and proportions of water removal substances in the water removal device on the peak area of specific components in standard gases. Every treatment group’s reported values are the averages of three independent replicates per filler component. Differences between treatments were determined by least significant differences, with *p* < 0.05. Different letters denote significant differences (*p* < 0.05) among the groups according to ANOVA followed by Duncan’s multiple range test.

Based on the standard gas, seven main compounds were detected: isoprene, trichloromethane, benzene, toluene, chlorobenzene, p-xylene, and pinene. Additionally, a comparative analysis was conducted to assess the influences of different water removal fillers (MgSO_4_, Na_2_SO_4_, MgSO_4_ + Na_2_SO_4_) on standard gas (Figure 2).

Compared with the standard gas without water vapor, it was observed that the concentrations of various compounds in the standard gas passing through the water were significantly reduced. The total content was 41.23% lower than that of the conventional standard gas (Figure 2). The results demonstrated that the presence of water in the detected gas has a significant impact on the detection results, and it is necessary to find a way to solve the water removal problem of the gas.

Next, various water removal materials were chosen as fillers to compare their respective effects on water removal, including Na_2_SO_4_, MgSO_4_, and MgSO_4_ + Na_2_SO_4_, respectively. After passing standard gas through three distinct water removal devices [DM(MgSO_4_), DM(Na_2_SO_4_) and DMm(MgSO_4_ + Na_2_SO_4_)], there was no significant alteration in the contents of various compounds compared to CK (Figure 2). The findings suggested that the utilization of Na_2_SO_4_, MgSO_4_, and MgSO_4_ + Na_2_SO_4_ as fillers had negligible effects on gas adsorption. Therefore, these fillers were chosen for the next step of the water removal experiment. Subsequently, when the standard gas passed through the water vapor and the DM(MgSO_4_) water removal device, the concentrations of various compounds were markedly decreased to 32.53% of those in the standard gas after passing through the DM(MgSO_4_) (Figure 2). For the DM(Na_2_SO_4_) removal device, the content detected from the standard gas through the water vapor decreased significantly to 22.47% of that from the standard gas, and for the DMm(MgSO_4_ + Na_2_SO_4_) water removal device, the reduction detected from the standard gas through the water vapor was even more substantial at 5.80% of that from the standard gas. The contents of various compounds detected in the standard gas through the water vapor using the DM(MgSO_4_), DM(Na_2_SO_4_), and DMm(MgSO_4_ + Na_2_SO_4_) water removal devices were significantly higher than those in the CK treatment group through the water vapor. However, they were lower compared to the content observed in the standard gas utilizing DM(MgSO_4_), DM(Na_2_SO_4_), and DMm(MgSO_4_ + Na_2_SO_4_), respectively. The findings indicated that the three water removal devices had different water removal effects. The water removal efficiency of DMm(MgSO_4_ + Na_2_SO_4_), which used the combination of MgSO_4_ + Na_2_SO_4_ as the water removal device filler, was the best.

### 3.2. Effect of Filling Type of Screening Filler on Water Removal Effect

**Figure 3:**
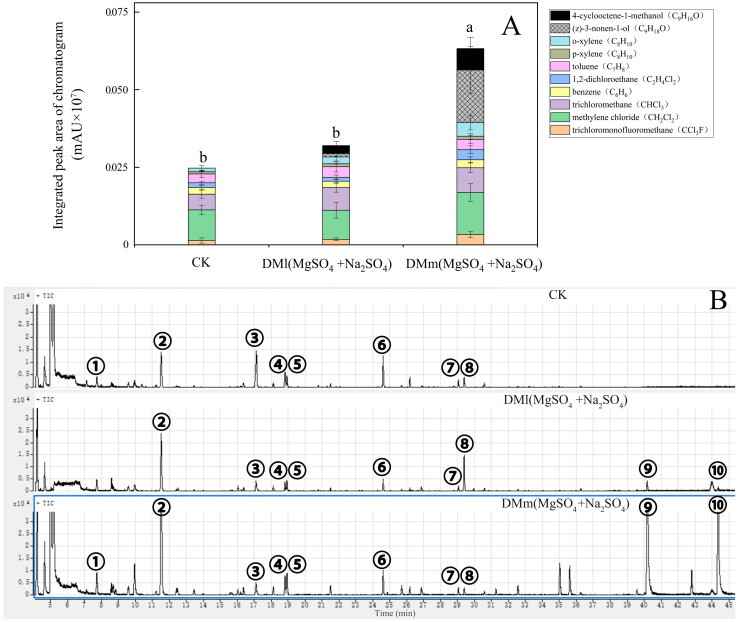
Effects of two water removal fillers (MgSO_4_ + Na_2_SO_4_) under two types of layered filling and mixed filling on forest BVOCs emission. (**A**) comparison of various components of BVOCs emitted from forests; (**B**) chromatogram of various components of BVOCs. The numbers in the chromatogram represent the following compounds: ①—trichloromonofluoromethane (CCI_3_F); ②—methylene chloride (CH_2_C1_2_); ③—trichloromethane (CHCI_3_); ④—benzene (C_6_H_6_); ⑤—1.2-dichloroethane (C_2_H_4_C1_2_); ⑥—toluene (C_7_H_8_); ⑦—p-xylene (C_8_H_10_); ⑧—o-Xylene (C_8_H_10_); ⑨—(z)-3-nonen-1-ol (C_9_H_18_O); and ⑩—4-cyclooctene-1-methanol (C_9_H_16_O). Every treatment group’s reported values are the averages of three independent replicates per filler component. Differences between treatments were determined by least significant differences with *p* < 0.05. Different letters denote significant differences (*p* < 0.05) among the groups according to ANOVA followed by Duncan’s multiple range test.

Based on the research findings in Figure 2, water removal devices with layered and mixed filling types were tested in a forest environment using MgSO_4_ + Na_2_SO_4_ filling (Figure 3). Multiple components of BVOCs in the forest were detected, including trichlorofluoromethane, methylene chloride, trichlormethane, benzene, 1,2-dichloroethane, toluene, p-xylene, o-xylene, (z)-3-nonen-1-ol, and 4-cyclooctene-1-methanol. Compared with CK, the total BVOCs contents using the DMl(MgSO_4_ + Na_2_SO_4_) and DMm(MgSO_4_ + Na_2_SO_4_) water removal devices significantly increased by 29.45% and 97.68%, respectively (Figure 3). It is important to note that two new compounds, (z)-3-nonen-1-ol and 4-cyclooctene-1-methanol, were identified, constituting 26.81% and 10.93% of the total, respectively. The newly discovered compounds were polar molecules. In forests with high humidity, the presence of water molecules can cause polar BVOCs signals to be obscured and go undetected.

Due to the similar polar characteristics between water molecules and target polar-molecules, the detection instrument was interfered with by water molecules, causing the detection signal of the target molecule to be submerged. Therefore, a mixed-fill type dewatering device was found to be the best choice (Figure 3).

### 3.3. Screening and Improvement of Experimental Results

**Figure 4:**
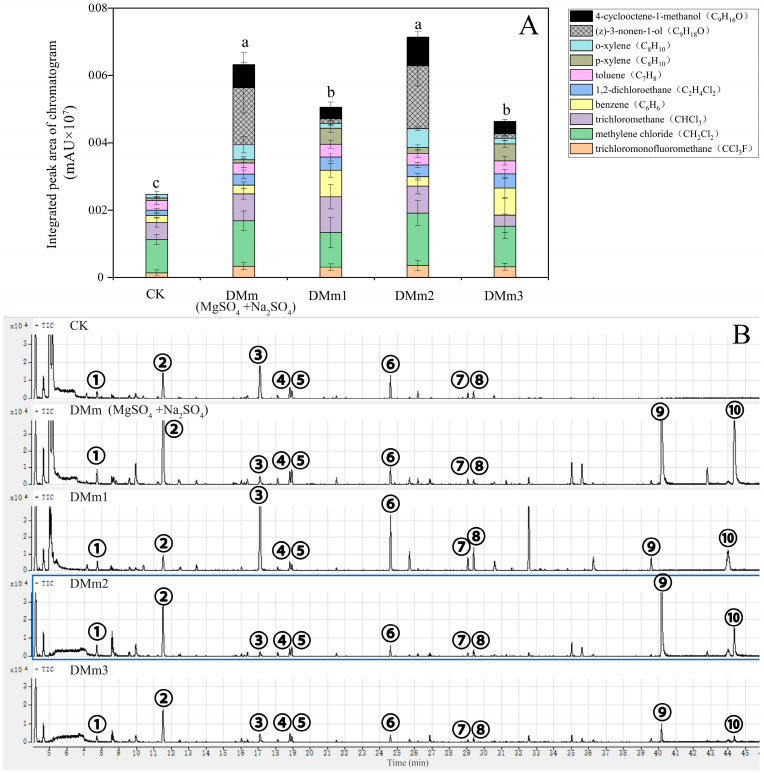
Effects of two water removal fillers (MgSO_4_ and Na_2_SO_4_) at different filling ratios on forest BVOCs emissions. (**A**) comparison of various components of BVOCs emitted from forest; (**B**) chromatogram of various components of BVOCs. The numbers in the chromatogram represent the following compounds: ①—Trichloromonofluoromethane (CCI_3_F); ②—Methylene chloride (CH_2_C1_2_); ③—trichloromethane (CHCI_3_); ④—benzene (C_6_H_6_); ⑤—1.2-dichloroethane (C_2_H_4_Cl_2_); ⑥—toluene (C_7_H_8_); ⑦—p-xylene (C_8_H_10_); ⑧—o-xylene (C_8_H_10_); ⑨—(z)-3-nonen-1-ol (C_9_H_18_O); and ⑩—4-cyclooctene-1-methanol(C_9_H_16_O). Every treatment group’s reported value is the average of three independent replicates per filler component. Differences between treatments were determined by least significant differences with *p* < 0.05. Different letters denote significant differences (*p* < 0.05) among the groups according to ANOVA followed by Duncan’s multiple range test.

According to the research results in Figure 2 and Figure 3, the dehydrated material MgSO_4_ + Na_2_SO_4_ was mixed and filled at ratios of 1:1 and 2:1. Additionally, we attempted to add a certain proportion of CuSO_4_ as an indicator, and the water removal devices were named DMm2 and DMm3, as listed in Table 1. Further experiments were performed in the stand to monitor the effect of the fill ratio on the type and content of BVOCs in order to optimize the fill ratio and determine the optimal filling ratio. The detected BVOCs in the forest, as shown in Figure 4, included trichlorofluoromethane, methylene chloride, trichlormethane, benzene, 1,2-dichloroethane, toluene, p-xylene, o-xylene, (z)-3-nonen-1-ol, and 4-cyclooctene-1-methanol. As for the addition of new compounds, which were polar molecules, their polarity allowed them to form strong interactions such as hydrogen bonds with water molecules, enabling them to be easily soluble in water. At higher humidity levels, polar molecules dissolve more easily, making them undetectable. Failure to remove water vapor in time would further reduce the sensitivity and accuracy of the detection.

Compared to CK, the total content of BVOCs increased by using the DMm(MgSO_4_ + Na_2_SO_4_), DMm1, DMm2, and DMm3 water removal devices, showing respective fold increases of 1.56, 1.04, 1.89, and 1.20 (Figure 4). Additionally, new compounds such as (z)-3-nonen-1-ol and 4-cyclooctene-1-methanol were also detected in the treatment groups using the water removal devices, accounting for 37.73%, 9.45%, 38.09%, and 9.25% of the total, respectively. Compared to the treatment group using the DMm(MgSO_4_ + Na_2_SO_4_) water removal device, the total BVOCs content of the treatment group using the DMm2, with the addition of CuSO_4_, increased by 23.97%. Similarly, compared to the treatment group using the DMm1, the total BVOCs content in the treatment group using the DMm3, with the addition of CuSO_4_, increased by 5.52% (Figure 4). The results indicated that CuSO_4_ not only exhibited water-absorbing characteristics, but also led to the formation of blue-hued hydrates upon moisture absorption, serving as a visual indicator for the real-time monitoring of moisture absorption in devices. Importantly, the addition of CuSO_4_ in the filler resulted in enhanced water removal device performance, improving both its water removal efficiency and its monitoring capacity. Therefore, the optimal filler for the water removal device consisted of a mixed filler of MgSO_4_ + Na_2_SO_4_ in equal proportions, combined with layered fillers using CuSO_4_ as a visual indicator, at a ratio of 3:3:1.

### 3.4. Verification Analysis and Application of Optimal Filling Conditions

According to the research results, the optimal filling device combination of MgSO_4_ + Na_2_SO_4_ mixed filling and CuSO_4_ layered filling (ratio of 3:3:1) was selected to collect BVOCs from mechanically damaged leaves of *Michelia maudiae* and *Cinnamomum camphora* tree species in order to further verify the water removal effect.

#### 3.4.1. Volatile Compound Collection and Validation Analysis of *Michelia maudiae* Tree Species

**Figure 5:**
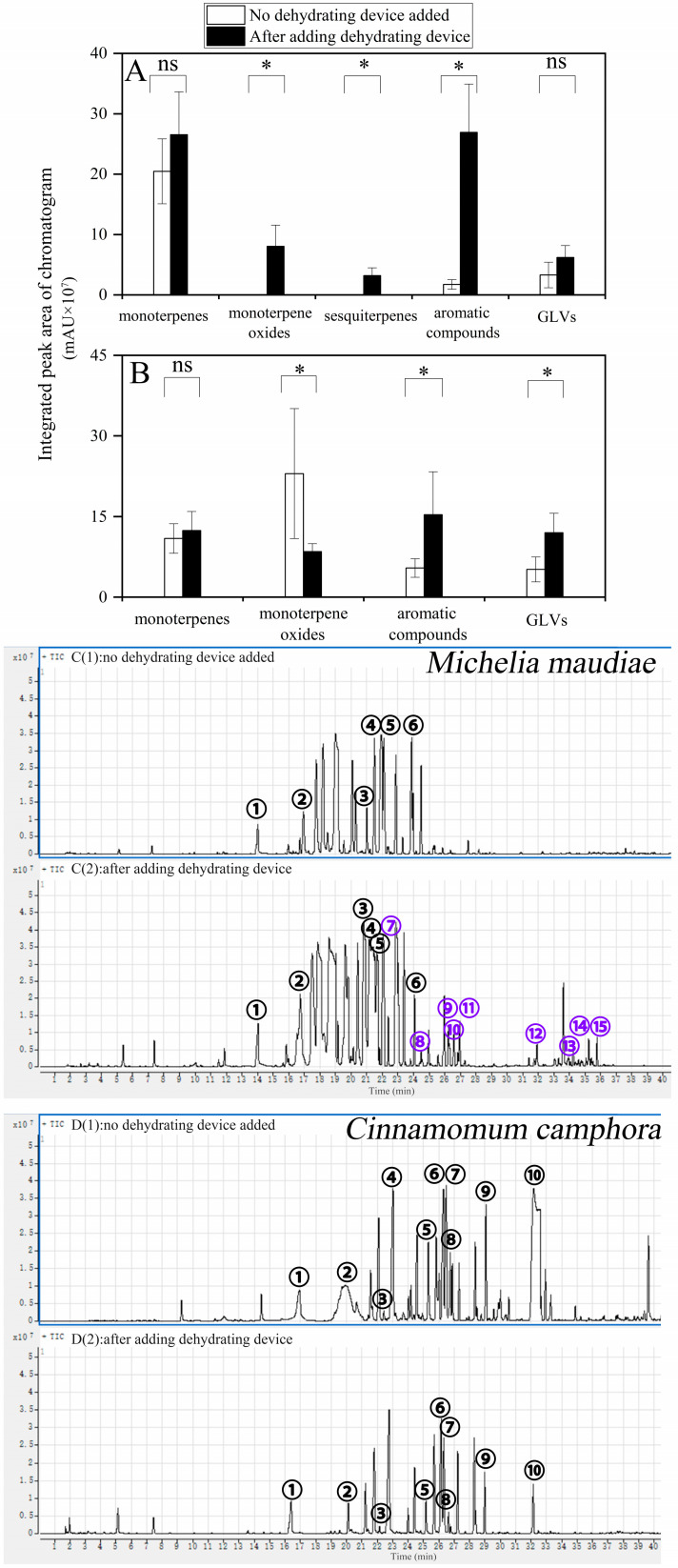
Effects of the water removal device on BVOCs from mechanically damaged leaves of *Michelia maudiae* and *Cinnamomum camphora* tree species. (**A**) Chromatogram of various components of BVOCs emitted from *Michelia maudiae*. (**B**) Comparison of various components of BVOCs emitted from *Michelia maudiae*. (**C**) Chromatogram of various components of BVOCs emitted no water removal device (**C1**) and adding water removal device (**C2**) from *Michelia maudiae*. The numbers in the chromatogram **C1** and **C2** represent the following compounds: ①—2-cyclohexen-1-ol (C_6_H_10_O); ②—geranyl propionate (C_13_H_22_O_2_); ③—α-phellandrene (C_10_H_16_); ④—cyclofenchene (C_10_H_16_); ⑤—β-pinene (C_10_H_16_); ⑥—pseudolimonene (C_10_H_16_); ⑦—eucalyptol (C_10_H_18_O); ⑧—fenchone (C_10_H_18_O); ⑨—isoborneol (C_10_H_18_O); ⑩—borneol (C_10_H_18_O); ⑪—α-terpineol (C_10_H_18_O); ⑫—copaene (C_15_H_24_); ⑬—γ-muurolene (C_15_H_24_); ⑭—β-guaiene (C_15_H_24_); and ⑮—isoleden (C_15_H_24_). (**D**) Comparison of various components of BVOCs emitted no water removal device (**D1**) and adding water removal device (**D2**) from *Cinnamomum camphora*. The numbers in the chromatogram **D1** and **D2** represent the following compounds: ①—2-cyclohexen-1-ol (C_6_H_10_O); ②—ethyl (2)-hex-3-enyl carbonate (C_9_H_16_O_3_); ③—2,2-dimethyl-5-methylenenorbornane (C_10_H_16_); ④—camphene (C_10_H_16_); ⑤—α-phellandrene (C_10_H_16_); ⑥—limonene (C_10_H_16_); ⑦—p-cymene (C_10_H_16_); ⑧—ocimene (C_10_H_16_); ⑨—δ-lsopropenyltoluene (C_10_H_12_); and ⑩—camphor-(C_10_H_16_O). Differences in average BVOCs emission rates both no water removal device and adding water removal device were also analyzes using paired-samples *t*-tests. Differences between treatments were determined by least significant differences with *p* < 0.05. “*” denotes significant differences between no-dehydrating and after adding dehydrating device leaf blade emission rates according to paired-samples *t*-tests (*p* < 0.05). “ns” denotes no significant differences. The blue number symbol “⑦–⑮” indicates new compounds emitted by damaged leaves after adding dehydrating device.

The results of the *Michelia maudiae* tree species showed that the total content of each category of compounds increased after adding a water removal device, among which monoterpenes, aromatic compounds, and green leaf volatiles (GLVs) increased by 29.73%, 146.07%, and 88.43%, respectively. Furthermore, emissions of new compounds, i.e., monoterpene oxides and sesquiterpenes, were detected (Figure 5A–D).

As shown in Table 2, the total content of monoterpenes increased by 29.73%, among which compounds such as α-phellandrene, cyclofenchene, β-pinene, and pseudolimonene increased significantly, with increases of 757.57%, 213.29%, 153.77%, and 146.73%, respectively. The aromatic compounds increased significantly, mainly geranyl propionate, which increased by 14.61 compared with the sample without adding a water removal device. GLVs also increased by 88.43%, primarily due to increases in 2-cyclohexen-1-ol and 3-pentanone with the detection of a new compound, (*E*)-2-octenal. Furthermore, emissions of new compounds, including monoterpene oxides and sesquiterpenes, were detected. The monoterpene oxides were mainly eucalyptol, fenchone, lsoborneol, borneol, and α-terpineol, while the sesquiterpenes were mainly copaene, γ-muurolene, β-guaiene, and isoleden. In general, the addition of a water removal device significantly increased the emission of volatile compounds from *Michelia maudiae*, leading to the detection of masked monoterpene oxides and sesquiterpenes.

#### 3.4.2. Volatile Compound Collection and Validation Analysis of *Cinnamomum camphora* Tree Species

Figure 5C,D shows the changes in the contents of various compounds after adding a water removal device. The results show that the contents of monoterpenes, aromatic compounds, and GLVs increased significantly, increasing by 13.48%, 183.46%, and 132.94%, respectively, compared with the sample without the dehydration device. However, the content of monoterpene oxides decreased. In addition, it can be observed from the chromatographic peak of Figure 5C that, after adding the dehydration device, the chromatographic peak shape appeared sharper and clearer, and the compounds covered by water could also be detected (Figure 5C,D).

As shown in Table 2, after the water removal device was installed, there was a collective increase in the content of monoterpene compounds, with specific compounds showing notable increases. Notably, ocimene, α-phellandrene, 2,2-dimethyl-5-methylenenorbornane, p-cymene, D-limonene, and camphene increased by 765.83%, 173.20%, 65.09%, 61.50%, 44.58%, and 17.24%, respectively.

However, in monoterpene oxides, the total content of compounds decreased. This was mainly reflected in camphor, which decreased by 92.05%. This was mainly due to the high water vapor content during forest sampling, resulting in poor separation of the integral peak shape. Aromatic compounds demonstrated a significant rise, mainly δ-lsopropenyltoluene, which increased by 138.27% compared with the sample without the addition of a water removal device. At the same time, the content of GLVs also increased significantly, primarily represented by the compounds ethyl (2)-hex-3-enyl carbonate and 2-cyclohexen-1-ol, which increased by 4.68 times and 1.36 times, respectively. In summary, the addition of a water removal device not only improved the separation efficacy of the chromatographic peaks, but also significantly enhanced the detection sensitivity and accuracy of various compounds.

## 4. Discussion

BVOCs are secondary metabolites produced during the normal growth and development of plants. Their emission amount accounts for about 90% of the total annual VOC emissions in the world [24,25], which are mainly composed of isoprene, monoterpene, sesquiterpene, etc., and more than 10,000 kinds of components have been found [26]. Some BVOCs are pharmaceutical and raw synthetic food materials, with health care, sterilization, air purification, human health-enhancing, and fatigue-relieving effects, which can achieve disease prevention and treatment [10,27]. There are also BVOCs that can have some negative effects on the environment, but the amounts released by plants are low. In recent years, studies have even shown that BVOCs play an important role in driving global atmospheric circulation and affecting climate change [9,28]. In view of this, the research on BVOCs is particularly important. Sampling and analysis of BVOCs is the basis and the most important part of research on BVOCs. During the sampling of BVOCs, researchers have observed that transpiration in plants leads to water vapor often being present along with BVOCs. This phenomenon is especially evident when BVOCs are collected in geographical locations close to water or in humid atmospheric environments, especially when BVOCs are collected in forest environments and plant leaves damaged by external factors [29,30]. This phenomenon reminds us of the need to consider the influence of water vapor on the results when analyzing and interpreting BVOC data, so as to more accurately assess the VOCs released by plants. Furthermore, during online analysis, the presence of water vapor causes column degradation and affects the ionization of molecular fragments, leading to qualitative discrepancies in the results obtained from MS detection [31,32]. This leads to undetected substances, qualitative errors, and inaccurate quantification. Due to the large amount of volatile organic matter present in BVOVs, in vivo sampling is usually accompanied by water vapor interference. Although commercial hydrophobic fillers are highly targeted, this method is only suitable for releasing a small amount of VOC, while BVOVs released by green leafy plants contain not only a large number of neutral organic substances, but also many biased organic substances. At present, there is a lack of effective strategies for removing water vapor to reduce interference in the sampling process of living green leafy plants. Considering the common problem, it is necessary to design a water removal device at the front end of the detection, which must be capable of removing water vapor without impacting the adsorption efficiency of gas collection, because BVOCs are easily absorbed and this could influence overall collection.

This experiment designed and optimized a plant BVOCs water removal device based on filler ratio, aiming to improve the accuracy and reliability of BVOCs analysis. In the design of the device, appropriate quartz glass tubes and various water removal fillers were selected, and the optimal filler ratio was determined through a series of experiments (Figure 2 and Figure 3). When collecting BVOCs in the forest, due to the frequent rainfall and high humidity in the southern region, BVOCs often become mixed with water vapor [33,34]. It is necessary to dewater BVOCs in the forest before detection. The process is essential in order to optimize the water removal device and ultimately improve the efficiency of BVOC collection in the forest environment, thereby improving the accuracy of the analytical data. The most preferred filler combination for the water removal device is the combination of MgSO_4_ + Na_2_SO_4_ mixed filler and CuSO_4_ layered filler, with a ratio of 3:3:1. Furthermore, plants also release BVOCs when they are damaged, and at the same time, they release water vapor [29,35]. In response to the common phenomenon, it is necessary to use a water removal device to remove water from BVOCs, and the water removal efficiency needs further testing and validation (Figure 5). In the verification test of BVOCs’ composition and content of mechanically damaged leaves of *Michelia maudiae* and *Cinnamomum camphora* tree species, the tested water removal device’s (MgSO_4_ + Na_2_SO_4_ mixed filling and CuSO_4_ layered filling combination, the ratio was MgSO_4_:Na_2_SO_4_:CuSO_4_ = 3:3:1) validity and reliability were further perfectly confirmed (Figure 5). When MgSO_4_ [20,21], CuSO_4_ [23], and Na_2_SO_4_ [18,19] were mixed with water vapor, they formed hydrates, which contain a certain amount of water molecules within the crystals. The process served to remove water vapor from BVOCs, thus achieving the purpose of water removal. It significantly improved the collection of volatile compounds, especially polar volatile compounds, and made the peak shape of the chromatogram clearer and sharper (Figure 4 and Figure 5). The successful detection of compounds previously masked by high moisture improved the comprehensiveness of sampling and the accuracy of the analysis, providing an important reference and insights for future sampling of plant volatile compounds.

In conclusion, the experiment successfully designed and optimized a plant BVOC water removal device based on the filler ratio. The combination of MgSO_4_ + Na_2_SO_4_ mixed filling and CuSO_4_ layered filling in a 3:3:1 ratio effectively enhanced the collection efficiency and accuracy of BVOCs. The method is time-saving, labor-saving, and low-cost, which is beneficial for the optimization of the research results. Next, the removal of water molecules from the water removal device by means of heating or desiccating agents needs to be considered in order to enable the device to be reused. This will be the focus of the future research.

## Figures and Tables

**Table 1:** Sample, water removal materials, proportions, and filling types of different water removal fillers. Single filling means filling one type of water removal filler into the device. Layered filling means filling different water removal fillers into the device in sequence according to certain proportions to form a layered structure. Mixed filling means mixing different water removal fillers in specific proportions and then filling them into the device.

Test	Sample Source	Name	Dehydration Materials (DM)			
			MgSO_4_	Na_2_SO_4_	CuSO_4_ (as an Indicator)	Type of Filling
Gas test	standard gas	CK	-	-	-	-
	standard gas through water vapor	CK	-	-	-	-
	standard gas	DM(MgSO_4_)	100%	0	0	single fill
	standard gas through water vapor	DM(MgSO_4_)	100%	0	0	single fill
	standard gas	DM(Na_2_SO_4_)	0	100%	0	single fill
	standard gas through water vapor	DM(Na_2_SO_4_)	0	100%	0	single fill
	standard gas	DMm(MgSO_4_ + Na_2_SO_4_)	50%	50%	0	mixed fill
	standard gas through water vapor	DMm(MgSO_4_ + Na_2_SO_4_)	50%	50%	0	mixed fill
Application test	forest	CK	-	-	-	-
	forest	DMl(MgSO_4_ + Na_2_SO_4_)	50%	50%	0	layered fill
	forest	DMm(MgSO_4_ + Na_2_SO_4_)	50%	50%	0	mixed fill
	forest	DMm1	66.67%	33.33%	0	mixed fill
	forest	DMm2	42.86%	42.86%	14.28%	mixed fill
	forest	DMm3	57.14%	28.57%	14.28%	mixed fill
	*Michelia maudiae* tree species	CK	-	-	-	-
	*Cinnamomum camphora* tree species	CK	-	-	-	-
	*Michelia maudiae* tree species	DMm2	42.86%	42.86%	14.28%	mixed fill
	*Cinnamomum camphora* tree species	DMm2	42.86%	42.86%	14.28%	mixed fill

**Table 2:** Effects of the water removal device on various detected compounds of BVOCs from mechanically damaged leaves of *Michelia maudiae* and *Cinnamomum camphora* tree species. All experiments were arranged in an independent complete design with three replicates. The values presented are the means ± SE of the three replicates.

Compounds	Molecular Formula	Name	Retention Time (min)	Integrated Peak Area *Michelia maudiae* (mAU × 10^7^)		Integrated Peak Area *Cinnamomum camphora* (mAU × 10^7^)	
				No Water Removal Device Added	After Adding Water Removal Device	No Water Removal Device Added	After Adding Water Removal Device
Monoterpenes	C_10_H_16_	cyclofenchene	21.56	21.86 ± 7.55	68.50 ± 5.37	7.05 ± 0.53	7.25 ± 2.36
	C_10_H_16_	2,2-Dimethyl-5-methylenenorbornane	22.42	23.79 ± 2.62	5.39 ± 0.25	0.76 ± 0.13	1.25 ± 0.21
	C_10_H_16_	Camphene	23.02	60.01 ± 4.78	44.18 ± 2.55	29.95 ± 7.36	35.11 ± 8.86
	C_10_H_16_	γ-Terpinene	27.32	14.11 ± 1.15	6.80 ± 2.55	14.28 ± 3.63	12.90 ± 5.64
	C_10_H_16_	Pseudolimonene	24.03	15.14 ± 4.56	37.35 ± 2.54	2.94 ± 1.02	2.95 ± 0.42
	C_10_H_16_	β-Pinene	21.67	8.22 ± 4.53	20.86 ± 7.66	9.57 ± 5.36	4.55 ± 3.62
	C_10_H_16_	α-Phellandrene	25.31	5.85 ± 0.78	50.16 ± 8.21	4.17 ± 2.33	11.39 ± 1.00
	C_10_H_16_	D-Limonene	26.29	18.81 ± 9.27	11.41 ± 5.24	20.14 ± 7.46	29.12 ± 2.37
	C_10_H_16_	Ocimene	26.88	1.44 ± 0.62	1.37 ± 0.33	0.69 ± 0.26	5.98 ± 0.79
	C_10_H_14_	p-Cymene	26.48	35.36 ± 7.83	19.42 ± 2.53	15.05 ± 8.67	24.15 ± 7.69
Monoterpene oxides	C_10_H_16_O	Camphor	32.18			80.21 ± 22.56	6.38 ± 0.85
	C_10_H_18_O	Eucalyptol	26.74		28.42 ± 5.27	2.36 ± 0.53	10.56 ± 4.71
	C_10_H_18_O	Fenchone	24.06		99.01 ± 2.54		
	C_10_H_18_O	lsoborneol	26.24		4.77 ± 1.09		
	C_10_H_18_O	Terpinen-4-ol	26.31		2.18 ± 0.96		
	C_10_H_18_O	endo-Borneol	26.60		5.03 ± 0.57	6.01 ± 0.86	
	C_10_H_18_O	trans-Verbenol	26.84		1.54 ± 0.85		
	C_10_H_18_O	α-Terpineol	26.96		5.31 ± 1.52	3.40 ± 0.96	
Sesquiterpenes	C_15_H_24_	(-)-Aristolene	31.40		0.86 ± 0.19		
	C_15_H_24_	Ylangene	31.73		0.70 ± 0.12		
	C_15_H_24_	copaene	31.91		2.79 ± 0.63		
	C_15_H_24_	Longifolene	33.05		1.20 ± 0.56		
	C_15_H_24_	Caryophyllene	33.61		11.97 ± 5.62		
	C_15_H_24_	γ-Muurolene	34.20		1.79 ± 0.24		
	C_15_H_24_	β-Guaiene	35.23		3.41 ± 0.21		
	C_15_H_24_	isoledene	35.76		2.79 ± 0.63		
Aromatic compounds	C_10_H_12_	δ-lsopropenyltoluene	23.39			6.44 ± 0.57	15.36 ± 8.08
	C_11_H_20_O_3_	(*E*)-Hex-3-enyl isobutyl carbonate	26.02			7.77 ± 1.03	
	C_13_H_22_O_2_	Geranyl propionate	16.74	1.73 ± 0.53	26.93 ± 12.35	2.04 ± 0.36	
GLVs	C_5_H_10_O	3-pentanone	5.386	0.70 ± 0.03	2.87 ± 0.76	0.12 ± 0.09	3.81 ± 0.96
	C_6_H_10_O	2-cyclohexen-1-ol	14.039	5.89 ± 2.30	9.66 ± 1.85	8.21 ± 0.57	12.06 ± 3.05
	C_8_H_14_O	(*E*)-2-octenal	16.529		6.11 ± 3.55		
	C_9_H_16_O_3_	ethyl (2)-hex-3-enyl carbonate	19.92			4.05 ± 1.07	23.00 ± 8.64
	C_9_H_14_O	2,4-Dimethylcyclohex-3-ene-1-carbaldehyde	24.92			0.91 ± 0.31	
	C_9_H_12_O	3,4-Dimethylbenzyl alcohol	23.03			12.49 ± 6.86	9.17 ± 1.86

## Data Availability

No new data were created or analyzed in this study. Data sharing is not applicable to this article.
